# Electron and proton transport in wheat exposed to salt stress: is the increase of the thylakoid membrane proton conductivity responsible for decreasing the photosynthetic activity in sensitive genotypes?

**DOI:** 10.1007/s11120-021-00853-z

**Published:** 2021-06-14

**Authors:** Ulkar Ibrahimova, Marek Zivcak, Kristina Gasparovic, Anshu Rastogi, Suleyman I. Allakhverdiev, Xinghong Yang, Marian Brestic

**Affiliations:** 1 Institute of Molecular Biology and Biotechnologies, Azerbaijan National Academy of Sciences, 11 Izzat Nabiyev, Baku, AZ 1073 Azerbaijan; 2 Research Institute of Crop Husbandry, Ministry of Agriculture of the Azerbaijan Republic, Baku, Azerbaijan; 3 Department of Plant Physiology, Slovak University of Agriculture, Nitra, Slovakia; 4 Laboratory of Bioclimatology, Department of Ecology and Environmental Protection, Poznan University of Life Sciences, Piątkowska 94, 60-649 Poznan, Poland; 5 Faculty of Geo-Information Science and Earth Observation (ITC), University of Twente, 7500 AE Enschede, The Netherlands; 6 K.A. Timiryazev Institute of Plant Physiology, Russian Academy of Sciences, Botanicheskaya Street 35, Moscow, 127276 Russia; 7 Institute of Basic Biological Problems, Russian Academy of Sciences, Pushchino, Moscow, 142290 Russia; 8 College of Life Science, State Key Laboratory of Crop Biology, Shandong Key Laboratory of Crop Biology, Shandong Agricultural University, Taian, People’s Republic of China; 9 Department of Botany and Plant Physiology, Faculty of Agrobiology, Food and Natural Resources, Czech University of Life Sciences, Prague, Czech Republic

**Keywords:** Wheat, Salt stress, Noninvasive measurements, Chlorophyll fluorescence

## Abstract

Effects of salinity caused by 150 mM NaCl on primary photochemical reactions and some physiological and biochemical parameters (K^+^/Na^+^ ratio, soluble sugars, proline, MDA) have been studied in five *Triticum aestivum* L. genotypes with contrasting salt tolerance. It was found that 150 mM NaCl significantly decreased the photosynthetic efficiency of two sensitive genotypes. The K^+^/Na^+^ ratio decreased in all genotypes exposed to salinity stress when compared with the control. Salinity stress also caused lipid peroxidation and accumulation of soluble sugars and proline. The amounts of soluble sugars and proline were higher in tolerant genotypes than sensitive ones, and lipid peroxidation was higher in sensitive genotypes. The noninvasive measurements of photosynthesis-related parameters indicated the genotype-dependent effects of salinity stress on the photosynthetic apparatus. The significant decrease of chlorophyll content (SPAD values) or adverse effects on photosynthetic functions at the PSII level (measured by the chlorophyll fluorescence parameters) were observed in the two sensitive genotypes only. Although the information obtained by different fast noninvasive techniques were consistent, the correlation analyses identified the highest correlation of the noninvasive records with MDA, K^+^/Na^+^ ratio, and free proline content. The lower correlation levels were found for chlorophyll content (SPAD) and *F*_v_/*F*_m_ values derived from chlorophyll fluorescence. Performance index (PI_abs_) derived from fast fluorescence kinetics, and *F*_735_/*F*_685_ ratio correlated well with MDA and Na^+^ content. The most promising were the results of linear electron flow measured by MultispeQ sensor, in which we found a highly significant correlation with all parameters assessed. Moreover, the noninvasive simultaneous measurements of chlorophyll fluorescence and electrochromic band shift using this sensor indicated the apparent proton leakage at the thylakoid membranes resulting in a high proton conductivity (gH^+^), present in sensitive genotypes only. The possible consequences for the photosynthetic functions and the photoprotection are discussed.

## Introduction

Being one of the extreme factors of the environment, soil salinity greatly impacts the development and productivity of agricultural plants (Arif et al. 2019; Rastogi et al. 2020). Millions of tons of agricultural products are lost every year due to soil salinization. As a result, people in many parts of the world suffer from food shortages. According to the FAO, over 6% of the world's land and more than 20% of cultivated land are affected by salinity, and by 2050, more than half of arable land will be unusable due to soil salinization.

High salinity causing osmotic, ionic, and oxidative stresses leads to plant death (Munns et al. 2006; Sharma et al. 2012; Zeeshan et al. 2020). Osmotic stress causes water deficiency manifested by weakening turgor, fading, stomatal closure, and cell growth cessation (Rengasamy 2006; Machado and Serralheiro 2017). On the other hand, it results in the excessive accumulation of toxic ions such as Na^+^ and Cl^−^ in the cell, which disrupts the normal course of metabolic processes. Salt stress also leads to oxidative stress caused by the formation of reactive oxygen species (ROS). In this case, changes in various components of the cell such as lipid peroxidation, reduced enzymatic activities, oxidation of proteins, and damage to DNA occur (Jena 2012; Gupta and Huang 2014; Isayenkov and Maathuis 2019; Rasel et al. 2020).

Large-scale scientific research is currently being carried out to obtain varieties with high adaptation ability to the effects of salt stress. Being a strategically important product, wheat occupies an essential place among cereals and other agricultural plants. Wheat manifests moderate tolerance to salinity. The rice is destroyed under 100 mM NaCl, whereas wheat continues to grow, but a loss is observed in the final yield (Munns 2005). In this regard, the study and choice of salt-tolerant and highly productive wheat genotypes and use them in the selection processes as parental forms are of great importance. To make breeding more efficient, fast, and noninvasive methods to screen the effects of stress are needed.

In addition to various tools to measure chlorophyll content (Kalaji et al. 2017), there are multiple methods to determine the functional state of photosynthetic apparatus based on chlorophyll fluorescence before visual detection of the plant's damage can be applied (Brestic and Zivcak 2013; Kalaji et al. 2016, 2018). One of the most rapid is the analysis of fast fluorescence (Strasser et al. 2004; Stirbet and Govindjee 2011), which was previously found to be extremely useful to detect the effects of multiple stresses (Pšidová et al. 2018), including salinity (Rastogi et al. 2020). The JIP-test analysis provides valuable information on the status and function of PSII photochemistry. However, the method has some limitations, given the measurements realized in dark-adapted samples using leaf clips (Stirbet et al. 2020). More direct information is provided using the PAM analysis of the chlorophyll fluorescence (Schreiber et al. 1986), but the measurements are slower. The low-cost multisensor technologies, such as PhotosynQ, were introduced to eliminate disadvantages, applying the standard PAM procedures using fast protocols in light-adapted samples in situ (Kuhlgert et al. 2016). Up to now, there is insufficient information how these measurements are reliable and useful. In addition to measurements of fluorescence induction, useful information on the stress effects can be obtained also by analysing the changes of fluorescence spectra using full spectra analyses (Cherif et al. 2010) or focusing on crucial spectral bands using multispectral approaches (Lichtenthaler and Babani 2004). The last-mentioned approach is more efficient and requires a lower cost of the devices. A great advantage of the spectral fluorescence measurements is the proximal sensing, i.e., no need of contact of the device with plants, with great potential to be used in automated systems (Humplík et al. 2015). One of the successful commercial systems was introduced by Cerovic et al. (2002) with various applications (Tremblay et al. 2011; Zivcak et al. 2017), including sensing of the stress effects (Bürling et al. 2013).

In this respect, the present research aimed to compare the standard indicators of salt stress effects and responses in five wheat genotypes having contrasting drought tolerance with the parameters obtained by the rapid measurements using four different noninvasive techniques. In addition to detecting the stress level, we also assessed the possibilities to use the new sensors to provide additional information on mechanisms of salt stress effects on photosynthetic electron and proton transport in plants of genotypes differing in salt tolerance.

## Materials and methods

### Experimental setup

Five winter wheat (*Triticum aestivum* L.) genotypes with contrasting drought tolerance (based on grain yield) known as Mirbashir 128 (MIR; tolerant), Gobustan (GOB; tolerant), Gyzyl Bughda (GYZ; tolerant), Fatima (FAT; sensitive), and Zirva 80 (ZIR; sensitive) genotypes from the GeneBank of the Azerbaijan Research Institute of Crop Husbandry have been used in the experiments. Seeds were sterilized for 15 min using a 10% SAVO solution (Biochemie, 124 Bohumin, Czech Republic), which contained 5% NaClO and washed three times with distilled water. Seeds were germinated on wet filter paper (Whatman® 3) in Petri dishes under 14 h/10 h (day/night) regime with 150 μmol m^−2^ s^−1^ light intensity at 24/18 °C. The 7-day-old seedlings having similar sizes were selected and transferred to 7 l plastic trays with an aerated (regularly six times a day for 2 h) Reid York nutrient solution. The nutrient solution was changed every 3 days. Plants were cultivated under 14-h photoperiod, at a temperature of 24/18 °C, relative humidity 55–60%, and light intensity 150 μmol m^−2^ s^−1^. Salt treatment was started after the emergence of the 3rd leaf by adding 150 mM NaCl. The salt was added only one-time; clean water was used daily to balance the water presence after evaporative loss in plant seedlings. The duration of salt treatment was 14 days.

### Determination of potassium and sodium ions

Potassium and sodium ions were determined using a PFP7 (Jenway-2007, England) flame photometer. First, the plant samples were combusted according to the method described by Allen et al. (1986). A mixture of sulphuric and perchloric acids (3:1) was added to 0.2 g of powdered plant samples and stored for a day. The next day, the test tubes were gradually heated to 250–270 °C in an oven. After combustion, the bleached solution in the test tube was cooled and used to determine sodium and potassium ions. Standard solutions of KCl and NaCl salts were used to construct the calibration curve.

### Determination of malondialdehyde

The samples' MDA content was determined by the reaction of thiobarbituric acid (TBT; Kumar and Knowles 1993). The homogenate obtained after crushing 0.5 g of leaves in 5% trichloroacetic acid (TCA) was precipitated at 1000 × *g* for 10 min. Then, 4 ml of a mixture of 0.5% TBT and 20% TCA was added to the supernatant. After heating in a water bath for 30 min, the mixture was transferred to ice (ice bath), cooled, and precipitated at 1000 × *g* for 15 min. The supernatant's optical density was measured at 532 and 600 nm using a Hitachi 557 spectrophotometer (Hitachi High-Tech Corporation, Japan).

### Determination of soluble sugar content

Sugars were determined by the anthrone–sulphuric acid method (Fales 1951). After adding 10 ml of 80% ethanol on 0.1 g of dried leaves, the obtained mixture was shaken in a shaker for 24 h. The homogenate was precipitated at 5000 × *g* for 10 min, 2.5 ml of anthrone was added to the supernatant (0.5 ml) and heated in a water bath at 40 °C for 30 min. Anthrone reagent was prepared by dissolving 0.2 g anthrone in a 100 ml concentrated H_2_SO_4_. After cooling, the optical density of the mixture was measured at 625 nm using a Hitachi 557 spectrophotometer (Hitachi High-Tech Corporation, Japan). Sucrose was used to construct the calibration curve.

### Determination of proline content

Proline was determined in plants based on the Bates method (Bates et al. 1973). Ninhydrin (1.25 g) and cold crystalline acetic acid (30 ml) were pre-mixed and then dissolved in 20 ml of 6 M phosphoric acid. The mixture was kept in cold (4 °C) for a max of 24 h. After the homogenization of 0.5 g of the plant material in 10 ml of 3% sulfosalicylic acid, it was filtered through two layers of Whatman paper. Then 2 ml of ninhydrin and 2 ml of acetic acid were added, and the mixture was boiled at 100 °C for 1 h. Immediately after boiling, the reaction was stopped by transferring to a water–ice mixture, 4 ml of toluene solution was added, and the mixture was shaken for 15–20 s. After the storage at room temperature, until the two-phase system was fully formed, the optical density of the organic-toluene phase containing chromophore was measured using a Hitachi 557 spectrophotometer (Hitachi High-Tech Corporation, Japan), at 520 nm. Toluene solution was taken as standard. The proline concentration was determined by the calibration curve and calculated on a fresh weight basis.

### Measurements of rapid chlorophyll *a* fluorescence kinetics in the dark-adapted state

Chlorophyll *a* fluorescence was measured by a portable non-modulated fluorimeter Plant Efficiency Analyser (Handy PEA; Hansatech Instruments, Kings Lynn, UK). Chlorophyll *a* fluorescence measurements were performed on both control and stressed plants after the third day of salt treatment during the experiment. Intact flag leaves of wheat plants were adapted to darkness for 30 min using light-withholding clips. After the adaptation of leaves to darkness, a single strong 1 s-light pulse (3500 µmol m^−2^ s^−1^) was applied to them with the help of three light-emitting diodes (650 nm). The fast fluorescence kinetics (*F*_0_ to *F*_M_) was recorded from 10 µs to 1 s. For each variety and treatment, at least ten repetitions were applied. The measured data were used to calculate the JIP-test parameters using Biolyzer v. 3.06 HP software (Strasser et al. 2010; Kalaji et al. 2016). Among different photosynthetic parameters, maximal photochemical efficiency of PSII (*F*_v_/*F*_m_) and performance index (PI_abs_), non-photochemical quenching (NPQ) were selected to be represented in this manuscript due to their proven sensitivity to identify the salt stress response in wheat genotypes. *F*_v_/*F*_m_ indicates the maximum quantum yield of PSII photochemistry, whereas PI_abs_ is a photosynthetic parameter connected to a number of different phenomena related to PSII photochemistry (Živčák et al. 2008; Kalaji et al. 2017).

### Measurements of electron and proton transport-related processes in light

Electron transport rate (ETR_PSII_), electrochromic bandshift, and photosystem I activity assessments were performed using a handheld MultispeQ V2.0 device linked to the PhotosynQ platform (Kuhlgert et al. 2016; www.photosynq.org). The protocol is available on the PhotosynQ platform under the name Photosynthesis Rides 500 (https://photosynq.org).

Fluorescence-based ETR_PSII_ was calculated using the values of PSII quantum yield (*Φ*_PSII_) obtained by a pulse–amplitude modulation (PAM) method at photosynthetically active radiation (PAR) of 500 μmol photons m^−2^ s^−1^. After considering the leaf absorbance changes causing relatively small effects on the obtained ETR_PSII_ values, we calculated the electron transport rate using the basic formula: ETR_PSII_ = 0.84 * 0.5 * *Φ*_PSII_ * PAR (Krall and Edwards 1992).

In parallel with the fluorescence measurements, the number of active PSI centers was assessed as the maximum amplitude of the P700 signal estimated by absorbance shifts at 820 nm measured under steady-state and rapid-saturating light and far-red pulses (Schreiber and Neubauer 1987; Harbinson and Woodward 1987).

The total electrochromic shift (ECS_t_) during light–dark transitions was determined by the absorbance change at 525 nm induced by 300 ms of dark intervals. The proton motive force was estimated on dark-adapted leaves after different illumination periods (500 μmol s^−1^ m^−2^ at 650 nm), as the total amplitude of the light–dark ECS_t_.

The thylakoid conductivity to protons (gH^+^) was determined by the dark interval relaxation kinetics (DIRK) of the electrochromic shift (ECS) at 520 nm (Avenson et al. 2005; Takizawa et al. 2007) and was utilized as a measure for the chloroplast ATP synthase activity. The proton motive force (pmf) was assessed as the amplitude of the first-order decay kinetics of the ECS trace in the first 300 ms. The product of ECS_t_ * gH^+^ was used as an estimate of the proton transport through the thylakoid membrane, which may be related to the rate of ATP synthesis (Kanazawa and Kramer 2002; Kanazawa et al. 2017).

### Measurements of chlorophyll fluorescence ratio

The ratio of red/far-red fluorescence (SFR, *F*_735_/*F*_685_) was assessed using a noninvasive portable optical fluorescence sensor Multiplex-3® (Force-A, Paris, France). The leaves were measured by proximal sensing under ambient light conditions (Mbarki et al. 2018).

Chlorophyll fluorescence ratio was calculated from values of fluorescence measured at 735 nm (FRF) and 685 nm (RF) after excitation by red light (635 nm), as follows:

$$F_{735} /F_{685} = {\text{SFR}} = {\text{FRFR}}/{\text{RFR}}.$$

Because the diameter of the measuring area was only 50 mm, 6–7 measurements were taken on each plant in different positions to account for heterogeneity in leaf color and structure.

### Statistical analyses

Statistical analysis was performed using two-way analysis of variance (ANOVA) followed by the post hoc Tukey HSD test (*P* < 0.05) using the Statistica version 9.0 software (Statsoft, Inc., Tulsa, Oklahoma, USA). The factors analyzed were Salt (irrigated vs. non-irrigated variant) and Genotype (6 genotypes). The data presented in graphs represent the mean value ± standard error. Ten individuals of each genotype were analyzed using noninvasive methods. The mutual correlations between the values of the contents of phenolic compounds or calculated parameters determined for individual plants were assessed using the matrix of values of the Pearson's correlation coefficients (*r*) and statistical significance obtained by the statistical functions of Statistica version 9.0 software (Statsoft, Inc., Tulsa, Oklahoma, USA). The correlations were classified according to Cohen (1988), with values higher than 0.7 were considered as a good correlation.

## Results

**Fig. 1:**
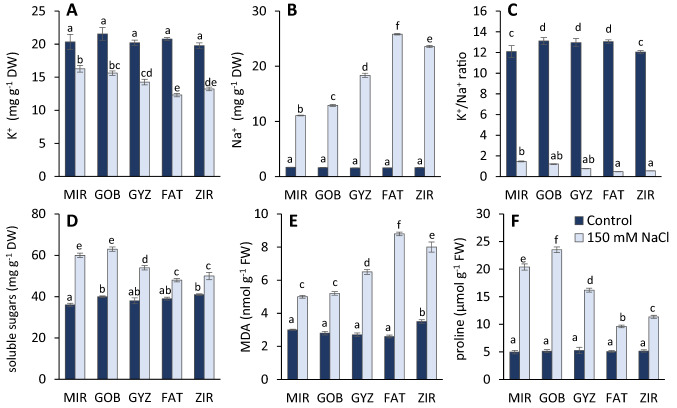
Effect of salinity on potassium (K^+^) content (**A**), sodium (Na^+^) content (**B**), K^+^/Na^+^ ratio (**C**), soluble sugars (**D**), MDA content (**E**), and proline content (**F**) in different wheat genotypes (*MIR* Mirbashir 128, *GOB* Gobustan, *GYZ* Gyzyl Bughda, *FAT* Fatima, *ZIR* Zirva 80). All data are presented as the mean value ± standard error (SE). Data in columns with the different letters are significantly different according to Duncan's multiple range test at *P* = 0.05

NaCl caused a decrease in the amount of K^+^ and an increase in Na^+^ in the leaves of the studied genotypes (Fig. 1A, B). The highest value of the K^+^/Na^+^ ratio in stress-exposed plants was recorded in the MIR, GOB genotypes, and the lowest value was recorded in the FAT and ZIR genotypes (Fig. 1C).

150 mM concentration of NaCl caused a sharp increase in proline and soluble sugars concentration (Fig. 1D, F). The proline amount was higher by 4.1–4.6-fold in the MIR, GOB genotypes, and 1.9–2.2-fold higher in the FAT and ZIR genotypes, respectively, compared with the control. The content of soluble sugars increased by 1.6–1.7-fold in the MIR and ZIR genotypes and 1.2-fold in the FAT and ZIR genotypes. MDA is a secondary metabolite and is an indicator of lipid peroxidation, which causes damage to membranes. An increase in the MDA content was observed after NaCl treatments in each of the five genotypes. In the drought-sensitive genotypes, the MDA content was 2.3–3.4-fold higher than in control. In tolerant varieties, this increase was not more than 1.6-fold (Fig. 1E).

**Fig. 2:**
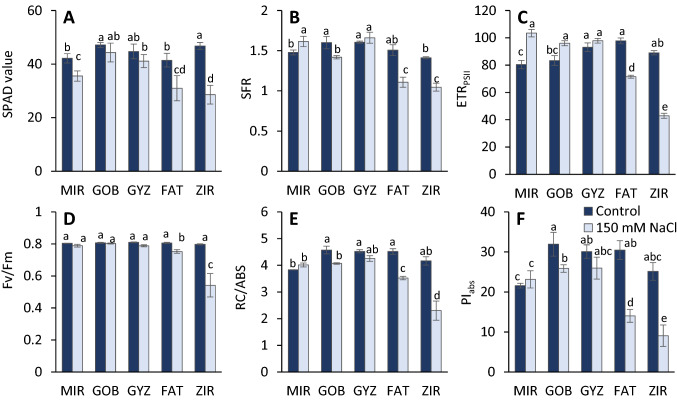
Effect of salinity on the values of parameters derived from noninvasive measurements of leaf absorbance and fluorescence: chlorophyll content measured in SPAD values (**A**), fluorescence ratio *F*_735_/*F*_685_ derived from multispectral fluorescence records (**B**), rate of linear electron transport calculated from the fluorescence quenching analysis in the light-adapted state (**C**), the maximum quantum yield of PSII photochemistry (**D**), reaction centers per absorbed light unit: RC/ABS (**E**), and Performance Index: PI_abs_ (**F**) derived from the analysis of fast fluorescence kinetics in dark-adapted leaves of different wheat genotypes (*MIR* Mirbashir 128, *GOB* Gobustan, *GYZ* Gyzyl Bughda, *FAT* Fatima, *ZIR* Zirva 80). All data are presented as the mean value ± standard error (SE). Data in columns with the different letters are significantly different according to Duncan's multiple range test at *P* = 0.05

The measurements using the chlorophyll meter (Fig. 2A) indicate a decrease in chlorophyll concentration in salinity treated plants, with the highest decrease (~ 33%) in ZIR. The multispectral fluorescence proximal sensing showed a significant decrease of the fluorescence ratio *F*_735_/*F*_685_ by 25% in the FAT and ZIR genotypes, whereas MIR and GYZ genotypes have shown a slight increase in multispectral fluorescence records (Fig. 2B). The rate of linear electron transport calculated from the fluorescence quenching analysis in the light-adapted state (ETR_PSII_) increased or was not affected in salinity tolerant genotypes, whereas it decreases for FAT and ZIR (Fig. 2C).

The fast fluorescence kinetics measurements in a dark-adapted state indicated that the maximum quantum yield of PSII photochemistry (*F*_v_/*F*_m_) slightly decreased in sensitive genotypes FAT and ZIR (Fig. 2D). A significant decrease in the number of active PSII reaction centers (RC/ABS) and performance index value (PI_abs_) was observed in a majority of the genotypes analyzed, except for MIR (Fig. 2E, F).

**Fig. 3:**
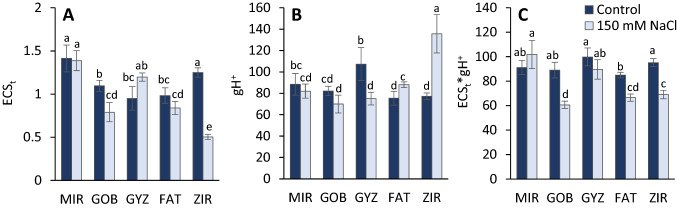
Effect of salinity on the values of parameters derived from noninvasive measurements of the dark interval relaxation kinetics (DIRK) of leaf absorbance signal at 520 nm (electrochromic bandshift): maximum amplitude of ECS signal: ECS_t_ (**A**), proton conductance of chloroplast ATP synthase: gH^+^ (**B**), estimated proton flux through thylakoid lumen calculated as a product of ECS_t_ and gH^+^: ECS_t_ * gH^+^ (**C**). The parameters were derived from the data obtained by the measurements in light-adapted leaves of different wheat genotypes (*MIR* Mirbashir 128, *GOB* Gobustan, *GYZ* Gyzyl Bughda, *FAT* Fatima, *ZIR* Zirva 80). All data are presented as the mean value ± standard error (SE). Data in columns with the different letters are significantly different according to Duncan's multiple range test at *P* = 0.05

The additional analysis focused on the proton transport at the thylakoid membrane was performed using electrochromic bandshift analysis at 520 nm, using the dark-interval relaxation kinetics analysis (Fig. 3). The maximum amplitude of the signal (ECS_t_) as a measure of the proton motive force significantly decreased in the most sensitive genotype under salt stress treatment and in genotypes GOB and FAT. In turn, we observed an increase in GYZ (Fig. 3A). In the proton conductance (gH^+^) case, we observed a decrease or no change in the group of the tolerant genotypes, whereas an increasing trend in the sensitive group (Fig. 3B). The estimate of the proton transport (ECS_t_ * gH^+^) indicated negligible effects (in MIR and GYZ) or the significant decrease of the proton flux (in GOB, FAT, and ZIR) due to the salt stress (Fig. 3C). Also, the effect of salinity was significant in all three parameters, and the values did not follow the grouping on the salt-sensitive and salt-tolerant genotypes evident from the basic indicators of the salinity effects (Fig. 1) or fluorescence-based indicators (Fig. 2).

The correlation analysis was performed to compare the reliability of the main parameters in estimating the salt stress effects on the photosynthetic apparatus (Table 1).

**Table 1:** Values of correlation indices for the relationships between the indicators obtained by the laboratory chemical analyses and the parameters derived from the noninvasive measurements by three different devices measured in salt-exposed plants

Device parameter	Handy PEA			PhotosynQ	Multiplex-3	SPAD-502
	*F*_v_/*F*_m_	PI_abs_	RC/ABS	ETR_PSII_	*F*_735_/*F*_685_	SPAD
K^+^	0.39^ns^	0.49*	0.49*	0.65**	0.60**	0.49*
Na^+^	− 0.54*	− 0.67**	− 0.64**	− 0.77**	− 0.70**	− 0.56*
K^+^/Na^+^	0.52*	0.59**	0.57**	0.74**	0.63**	0.47*
Proline	0.55*	0.65**	0.66**	0.73**	0.64**	0.64**
MDA	− 0.57**	− 0.72**	− 0.69**	− 0.77**	− 0.76**	− 0.66**
Soluble sugars	0.45*	0.56*	0.54*	0.67**	0.54*	0.60**

Bold font indicates high correlation

**Significant at the *P* < 0.01 level; *significant at the *P* < 0.05 level; ^ns^non-significant

The analysis showed a good relationship between biochemical and noninvasive parameters, especially in the electron transport rate measured by the MultispeQ V2.0 device, ratio *F*_735_**/***F*_685_ derived from the proximal sensing of multispectral fluorescence using Multiplex-3 device and performance index (PI_abs_) analyzed by Handy PEA. The correlation in these parameters was higher than the most frequently used chlorophyll content analyses or *F*_v_/*F*_m_ parameter calculated from the dark-adapted samples.

## Discussion

The effects of salinity and the traits associated with salinity tolerance have gained great attention from plant scientists. In this study, the set of genotypes well characterized in terms of their salinity tolerance was applied to compare the standard analytical and biochemical indicators with the multiple parameters obtained using noninvasive methods.

The leaf chemical analyses indicated the genotypic differences in accumulation of Na^+^ in leaves, with expected higher accumulation in sensitive genotypes (FAT and ZIR) compared to the tolerant group. The increase in Na^+^ was accompanied by a decrease in K^+^, and the high value of the K^+^/Na^+^ ratio was observed in the tolerant genotypes MIR and GOB based on grain yield, which is consistent with published results (Ghogdi 2012; Nieves-Cordones et al. 2016). Investigations related to the plant adaptation to salt stress are mainly focused on the transport system that maintains cellular homeostasis (Tahal et al. 2000; Almeida et al. 2017). The maintenance of ionic homeostasis through the absorption of ions and their compartmentalization in the cell ensures the plant's growth and development not only under favorable conditions but also under conditions of salt stress. Maintaining a high level of the K^+^/Na^+^ ratio in the cell is one of the necessary conditions for the normal functioning of the plant cell. Sodium is a toxic element, and its high concentration leads to the disruption of various metabolic processes in plants (Zafar et al. 2015; Mbarki et al. 2018; Rastogi et al. 2020). On the contrary, K is an essential element for plant growth and development and is involved in the maintenance of cell turgor, osmotic regulation, activation of enzymes participating in metabolism, and the synthesis of carbohydrates and proteins (Rahneshan et al. 2018).

Besides osmotic and ionic stresses, salinity also causes oxidative stress, which is accompanied by ROS formation. Highly reactive compounds such as singlet oxygen (^1^O_2_), superoxide anion (O_2_^−·^), hydrogen peroxide (H_2_O_2_) and hydroxyl radical (^·^OH) affect proteins, lipids, and nucleic acids in the cell disrupting their structure (Rastogi and Pospíšil 2010, 2012). Saturated fatty acids, which are among the main components of membrane lipids, undergo peroxidation by free radicals (Elkahoui et al. 2005). As a result of lipid peroxidation, membrane integrity is violated due to increased permeability (Elkahoui et al. 2005). Malondialdehyde is a lipid peroxidation product and is considered an indicator of oxidative damage (Rastogi et al. 2014). Therefore, MDA is considered the best salinity marker in plants and widely studied in stress-exposed plants. Lipid peroxidation was detected in various plants treated with NaCl, such as mustard (Ahmad et al. 2012), bean (Azooz et al. 2011), wheat, and barley (Ibrahimova et al. 2019; Zeeshan et al. 2020). Lipid peroxidation is more common in salt-sensitive species than in resistant ones (Koca et al. 2007; Ksouri et al. 2007). Our results are consistent with the results of previous studies (de Azevedo Neto et al. 2006; Keutgen and Pawelzik 2008; Falleh et al. 2012) and lipid peroxidation was found to be higher in the salinity sensitive FAT and ZIR genotypes. Based on the results of our research, the MIR and GOB genotypes have better protection against oxidative damage under salt stress.

Although there is a decrease in the rate of CO_2_ assimilation in plants exposed to abiotic stresses, including salinity, there are many studies on the accumulation of sugars (Murakeözy et al. 2003; Radi et al. 2013). Soluble sugars are osmolytes, the accumulation of which is induced under salt stress (Cheeseman 1988). Accumulation of sugars in the cell under stress regulates the osmotic balance between the cytosol and vacuoles, thereby ensuring the structural and functional stability of the macromolecules (Rhodes 1987; Smirnoff and Cumbes 1989; Chelli-Chaabouni et al. 2010). In our study, the accumulation of sugars was observed in the leaves of salt-exposed plants, and the increase was higher in the salt-tolerant genotypes. The increase in the content of sugars in plants is considered to be the result of osmotic regulation. Thus, the synthesis of sugars in both salt-tolerant and sensitive genotypes occurs in response to the effects of NaCl (Radi et al. 2013; Zheng et al. 2012).

Amounts of amino acids such as proline, asparagine, aminobutyric acid are involved in osmotic regulation and increase sharply when the plant is exposed to different abiotic or biotic stress factors. Synthesis and accumulation of proline are among the main indicators of stress-induced responses. In this regard, proline accumulation in the plant cells under salt stress is considered one of the main parameters in selecting salt-tolerant genotypes (Yildiz and Terzi 2013). Proline also possesses the properties of antioxidants that act as chaperones to protect the structure of macromolecules from destruction when the cell is dehydrated (Yan et al. 2013; Ashraf and Foolad 2007). Therefore, proline can act as an enzyme protectant, free radical scavenger, cytosolic pH buffer stabilizer for subcellular structures, and cell redox balancer (Verbruggen and Hermans 2008). In the presented study, 150 mM NaCl induced a sharp increase in the proline concentration, and this increase was more pronounced in tolerant varieties. These results are consistent with the previous studies (Tammam et al. 2008; Mbarki et al. 2018; Rahneshan et al. 2018; Rastogi et al. 2020).

The question, why the tolerant plants produce higher concentrations of osmolytes than the sensitive genotypes is very complex. There are several cellular mechanisms by which organisms alleviate the adverse effects of abiotic stresses. One of them is an accumulation of compatible solutes (Yancey et al. 1982; Burg and Ferraris 2008), providing osmotic adjustment, ROS detoxification, protection of membrane integrity, and enzymes/protein stabilization (Ashraf and Foolad 2007; Bohnert and Jensen 1996; Yancey 1994). Stress highly regulates the synthesis of osmolytes (e.g., proline, sucrose, polyols, trehalose, glycine betaine) in stress-tolerant species. According to multiple authors, in most plants exposed to salt stress proline accumulation is correlated with stress tolerance, and its concentration is higher in salt-tolerant plants compared to sensitives (Fougère et al. 1991; Gangopadhyay et al. 1997; Madan et al. 1995; Petrusa and Winicov 1997; Szabados and Savoure 2010). There are different stress-response signaling pathways such as MAP kinase signaling, calcium signaling, ABA signaling, and ROS signaling leading synergistically to accumulation of different osmolytes in prokaryotes and eukaryotes. In plants, there is the salt overly sensitive (SOS) pathway and the abscisic acid (ABA) stress signaling pathway responsible for the accumulation of osmolytes (proline, mannitol, and glycine betaine) (Zhu 2001; Ji et al. 2013). The SOS gene overexpression resulted in increased salt tolerance of plants (mainly tolerant plants), as it helped in the maintenance of ion homeostasis, and may helped the synthesis of compatible solutes (Yang et al. 2009).

Although the genotypic differences in expression of the genes responsible for individual protective mechanisms responsible for observed differences among the genotypes were not assessed in this study, the analytical (chemical and biochemical) parameters applied in this study provided a set of representative indicators covering multiple salt stress effects as well as mechanisms of tolerance at the different level, enabling to identify a high diversity of the responses in the set of tested genotypes, which could be efficiently compared with the results of the noninvasive indicators.

An increase in salinity typically leads to a decrease of chlorophyll and may serve as an indicator of salt stress tolerance in wheat genotypes (Sairam et al. 2005). Similar results are obtained for noninvasive measurements using chlorophyll meters (Munns and James 2003; El-Hendawy et al. 2007), and it was clearly shown that SPAD readings significantly correlated with the chlorophyll content measured in a destructive way (Kiani-Pouya and Rasouli 2014).

As the SPAD measurements have several technical disadvantages, such as a very low measured leaf area, time and labor-consuming manual measurements, possibilities of leaf damage, and some others, there are multiple attempts to replace this technique with some proximal or remote techniques based on spectral reflectance (Gitelson 2003) or chlorophyll fluorescence spectral ratios (Gitelson et al. 1999). In our study, we applied the proximal sensing of the *F*_735_/*F*_685_ ratio known as SFR (Simple Fluorescence Ratio) measured by Multiplex-3 (Force-A, France) device. In well-controlled conditions, the ratio excellently correlates with the chlorophyll content (Lichtenthaler and Babani 2004), as the red fluorescence centered near 685 nm emitted deeper inside the leaf tissue is reabsorbed by the chlorophyll more than the far-red Chl fluorescence (Buschmann 2007). However, it was also shown that the *F*_735_/*F*_685_ is sensitive to photosystem I to photosystem II ratio (Brestic et al. 2015), as the far-red fluorescence originates mostly from PSI, whereas red fluorescence mostly from PSII. Unlike a stable PSI fluoresce, the PSII fluorescence greatly variates in response to the state of PSII photochemistry (Butler and Kitajima 1975), which influences the *F*_735_/*F*_685_ ratio, especially in non-controlled conditions. Thus, the *F*_735_/*F*_685_ (SFR) measured in field conditions positively correlates with a leaf chlorophyll content, but the correlation index was found to be relatively low (Li et al. 2013) and indicating SFR as a chlorophyll index is not fully correct.

On the other hand, both decreases in chlorophyll content and the photosynthetic activity lead to the decrease of the *F*_735_/*F*_685_ ratio, which makes this parameter useful as an indicator of stress. The sensitivity to different stress effects was previously found in various experiments (Cherif et al. 2010; Rinderle and Lichtenthaler 1988; Georgieva and Lichtenthaler 2006). Our results (Fig. 2B) indicate that the *F*_735_/*F*_685_ ratio can be useful as a non-specific indicator of salt stress effects in wheat, as well. Moreover, a good correlation level (Table 1) together with proximal (distant) application makes this technique a good candidate for the automated applications in crop phenotyping.

Although the phenotyping prioritizes remote or proximal sensing, the information on photosynthesis processes coming from these techniques is rather rough and less precise. Therefore, there are also attempts to efficiently develop and utilize fast, precise, and cost-effective leaf-clip measurements. The fast fluorescence kinetics analyses were identified as very suitable for high-throughput phenotyping because of the chlorophyll fluorescence transient measurement speed. The measured signal provides valuable information on the performance of PSII and efficiencies of specific electron transport reactions in the thylakoid membrane (Stirbet et al. 2018).

The fast fluorescence kinetics was found to be very efficient in salt stress studies, and multiple specific PSII responses were identified, such as changes in PSII heterogeneity (Mehta et al. 2010a), damage at the donor side (Mehta et al. 2010b), and the acceptor side (Lu and Vonshak 1999). Typically, the O-J amplitude of the fluorescence transient is increased (Kalaji et al. 2016), indicating a decrease in electron transport efficiency from PSII to Q_A_ and Q_B_. Moreover, the decrease of the number of active PSII reaction centers (Mathur et al. 2012; Rastogi et al. 2020) and PSII quantum efficiency (Mehta et al. 2010b) contribute to the overall adverse effect of salt stress on to function of PSII, which is also reflected by a significant decrease of performance index value (PI_abs_) observed in multiple studies (Mathur et al. 2012; Misra et al. 2001), including the studies analyzing the effects of graduated salt concentrations (Rastogi et al. 2020).

**Fig. 4:**
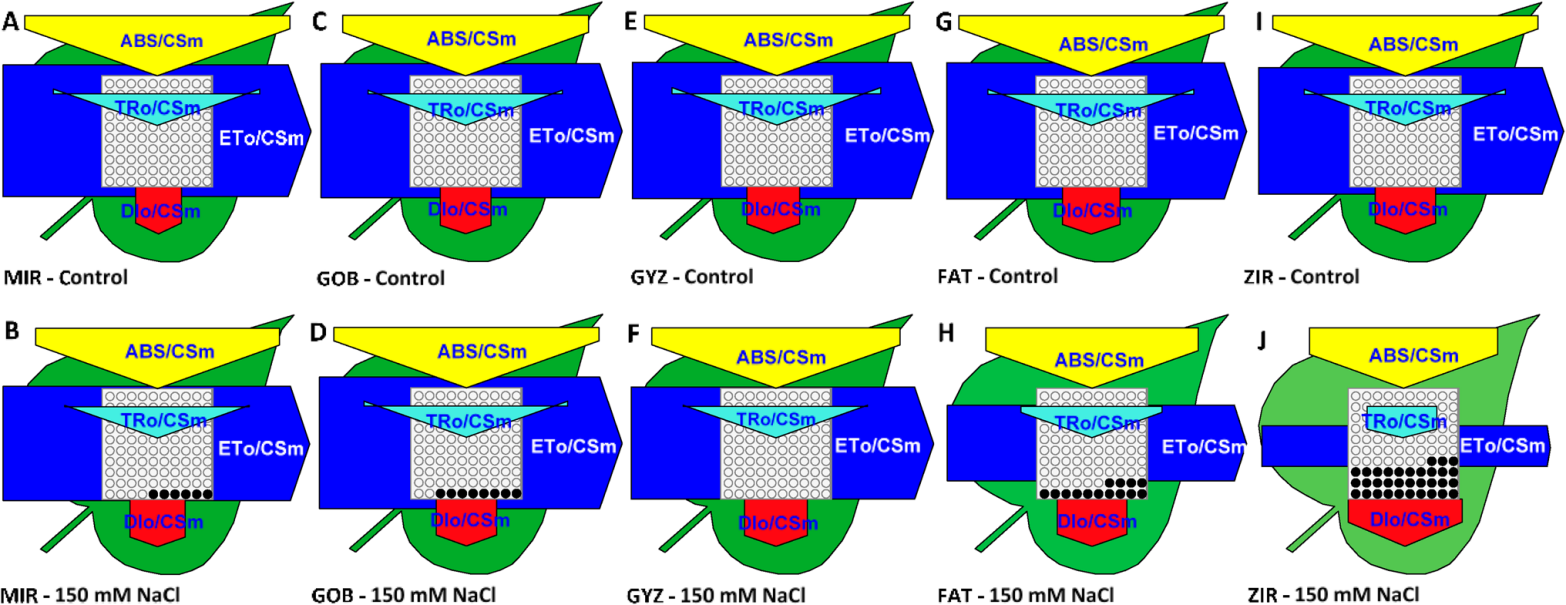
Phenomenological leaf models based on calculations of parameters derived from the analysis of fast fluorescence kinetics in dark-adapted leaves of different wheat genotypes (*MIR* Mirbashir 128, *GOB* Gobustan, *GYZ* Gyzyl Bughda, *FAT* Fatima, *ZIR* Zirva 80) in non-stressed (Control) and salt-stressed (150 mM NaCl) variants. The thickness of each arrow represents the mean value of absorbance (ABS/CS_m_), trapping flux (TR/CS_m_), electron transport (ET/CS_m_), or heat dissipation of excess light (DI/CS_m_), all expressed per leaf cross-section. The black points represent the fraction of inactive reaction centers. The models were generated using software Biolyzer 3.06 (Maldonado-Rodriguez, Laboratory of Bioenergetics, University of Geneva, Switzerland)

To visualize the changes in PSII photochemistry analyzed by the fast chlorophyll fluorescence transient using the model of Strasser et al. (2004), the phenomenological leaf models were created for all genotypes and variants (Fig. 4). It is evident that the changes in three tolerant genotypes were minor, with some visible limitation of electron transport at the PSII acceptor side (ET_o_/CS_m_) parameters and increase of the non-regulated energy loss (DI_o_/CS_m_). In two sensitive genotypes, we observed significant changes of all parameters, including substantial reduction of active reaction centers and decrease of all energy fluxes, including absorbed energy flux (ABS/CS_m_), trapping flux (TR_o_/CS_m_), and electron transport from PSII RCs to electron acceptors (ET_o_/CS_m_).

Our results are entirely in agreement with previous results, confirming the significant effects of salinity on PSII photochemistry. However, the observed effect was not uniform, but clearly genotype-dependent. The differences were found not only between the tolerant and sensitive group, but also the tolerant genotypes differed in responses to long-term salt treatment (Fig. 4). Moreover, the integrative parameter performance index (PI_abs_) was found to be a good indicator of salt stress effects, well correlating with the conventional indicators (Table 1).

Unlike to fast fluorescence transient measured in dark-adapted leaves, the steady-state PAM measurements enable estimating the rate of linear electron transport, which was closely correlated with the CO_2_ assimilation rate (Schreiber et al. 1995; Zivcak et al. 2013). However, compared to measurements of fast fluorescence transient, the PAM method has several disadvantages. First is the time needed to realize one measurement, which is more than one minute for PAM, compared to a few seconds needed to perform a one-second lasting measurement of fast chlorophyll fluorescence record (Brestic and Zivcak 2013). The second disadvantage is the high price of most PAM devices, limiting the option to solve the problem with speed by utilizing multiple instruments in parallel (Kalaji et al. 2017). To eliminate these two problems, a new low-cost sensor, MultispeQ connected to the open PhotosynQ network, was developed as a tool for large-scale plant phenotyping of plants (Kuhlgert et al. 2016). Multiple phenotyping studies with efficient utilization of fluorescence PAM records using MultispeQ sensor were published in the last period, such as analyses of drought stress effects in barley (Fernández-Calleja et al. 2020) or temperature stress in bean (Traub et al. 2018) and potato (Prinzenberg et al. 2018). The MultispeQ sensor was also used for phenotypic characterization of plants exposed to salts stress in *Arabidopsis thaliana* lines (Nepal et al. 2020) and potato (Prinzenberg et al. 2018) but without any attempts to assess the preciseness or reliability of the analysis compared to other methods or indicators. In our study, we identified sufficient sensitivity of the linear electron transport rate (ETR_PSII_) calculated from the PSII quantum efficiency to salts stress, as well as the significant differences between the genotypes (Fig. 1). Our results indicated that the ETR_PSII_ could be used to recognize the sensitive and tolerant genotypes. Comparing the parameters measured by different sensors (Table 1) identified the ETR_PSII_ parameter being the best correlating indicator based on the noninvasive measurements. It is a meaningful result considering the tight relationship between the effective PSII quantum efficiency and CO_2_ assimilation rate (Seaton and Walker 1990; Cheng et al. 2001; Zivcak et al. 2013).

Despite an increasing number of papers using the MultispeQ sensor, most applications are limited to the use of chlorophyll fluorescence only. However, the device was built as a multisensor designed for the simultaneous chlorophyll fluorescence measurements with several parameters based on measurements of light absorbance by the leaf at different wavelengths. In addition to estimation of chlorophyll content, the system is useful also for the assessment of the parameters closely related to the processes of electron and proton transport, such as the electrochromic bandshift measured at 525 nm, as well as the redox state of P700 based on absorbance changes at 820 nm (Kuhlgert et al. 2016). The dark interval relaxation kinetics (DIRK) of the electrochromic bandshift was found to be efficient in the assessment of the proton motive force (parameter ECS_t_) and the thylakoid conductivity to protons (parameter gH^+^), the product of which may serve as an estimate of proton flux (ECS_t_ * gH^+^) (Kanazawa and Kramer 2002; Avenson et al. 2005). The previous studies indicated a close relationship between the values of ECS parameters and efficient regulation of electron transport and photoprotection in stress (Zivcak et al. 2014; Brestic et al. 2016) or fluctuating conditions (Huang et al. 2018, 2019), including salts stress (Wu et al. 2019). However, all these studies were performed using expensive laboratory devices with limited use under field conditions. In turn, in a recent study, we applied the same DIRK protocol measuring ECS decay using a low-cost handheld device. Our results indicate the variations between the treatments as well as between the genotypes in all three parameters (Fig. 3). The ECS_t_ values were more-or-less following the trends observed by the chemical indicators or the fluorescence parameters, although the differences between the tolerant end sensitive genotypes were not clearly evident. A different situation was in gH^+^, as the proton conductance was significantly increasing in salt-exposed plants of the two sensitive genotypes, whereas the salt stress caused an expected decrease in salt-resistant genotypes (Fig. 4B). There are also differences in trends observed in estimates of electron transport rate (Fig. 2C) and the proton flux rate (Fig. 3C).

**Fig. 5:**
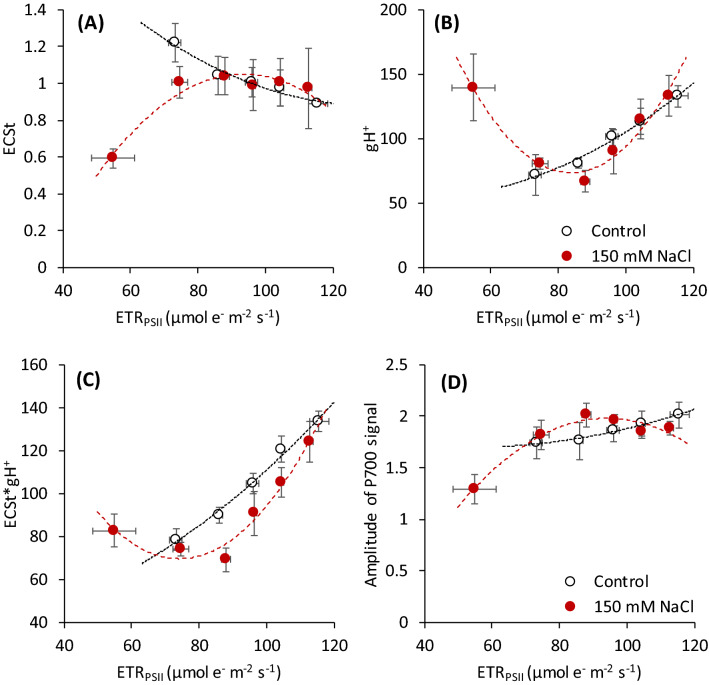
Relationship between the mean values of PSII electron transport rate (ETR_PSII_) and the parameters related to the proton transport and PSI activity analyzed simultaneously by the multisensor MultispeQ V2.0 (PhotosynQ, USA) in non-stressed (Control) and salt-stressed (150 mM NaCl) wheat plants. (**A**) The maximum amplitude of absorbance at 520 nm (ECS signal) measured with the far-red pulse and saturating light pulse, (**B**) proton conductivity of the thylakoid membrane through ATP synthase (gH^+^), (**C**) the proton flux estimated as a product of ECS_t_ and gH^+^, and (**D**) the amplitude of P700 signal measured as an absorbance signal at 820 nm. Data originate from the entire tested collection of wheat genotypes. The points represent the mean values of the samples in two variants sorted according to the ETR_PSII_ values into the six separate groups. The samples with very low ETR_PSII_ values (< 60 µmol e^−^ m^−2^ s^−1^) were present only in the salt-stress variant. Data are presented as the mean value ± standard error (SE) from 10 to 20 leaves

Electron transport rate (ETR_PSII_) was found to be well correlating with the stress indicators. Moreover, we can expect they correlate well with the overall photosynthetic performance of the measured leaves. Therefore, we used ETR_PSII_ as a reference value to compare the trends of the parameters derived from the absorbance data measured by the MultispeQ device in a group of non-stressed and salt-stressed plants (Fig. 5). As expected, there was a variation of the ETR_PSII_ values both in the control and stress groups. In non-stressed plants, electron transport variations may reflect different stomata openness and photosynthetic enzyme activation levels. The same factors may decrease the electron transport in salt-stressed plants, especially if the chloroplast are efficiently protected against toxic ions by their compartmentation (Robinson et al. 1983). However, there are also possible diverse effects of salt ions on chloroplast functions (Mehta et al. 2010b), well evident from the fast fluorescence analyses in sensitive plants (Fig. 3).

The correlation of ETR_PSII_ with ECS_t_ values in control plants (Fig. 5A) showed an inverse trend, where a decrease in electron transport rate led to an increase of ECS_t_. It was associated with the decrease of the proton conductance with decreasing PSII electron transport rate (Fig. 5B), resulting in a decrease of the proton flux clearly correlating with the decrease of the electron transport rate. These results are entirely expected, as the decrease in electron transport rate at the same light intensity increases energy-dependent non-photochemical quenching, which requires a decrease of the thylakoid lumen pH connected with an increase of ECS_t_ (Avenson et al. 2004; Kohzuma et al. 2009). As the decrease of assimilation rate indicated by a lower ETR_PSII_ is associated with lower demand for ATP, the decrease of gH^+^ is logical, probably resulting from ATP synthase downregulation (Kanazawa et al. 2017). The simultaneously measured chlorophyll fluorescence and ECS data confirm a clear correlation between the electron and proton transport previously shown by several studies (Avenson et al. 2004; Kohzuma et al. 2009). However, in the salt stress variant, these trends were followed only in samples with a high electron transport rate present in resistant genotypes but reversed in salt-stressed samples with a very low electron transport rate that occurred in sensitive ones. The sensitive plants had much lower ECS_t_ than expected according to a low ETR_PSII_, but, at the same time, they showed a high proton conductivity (gH^+^) resulting in a high proton flux, which was, evidently, not proportional to the observed electron transport rate. We previously observed a similar trend in wheat exposed to high temperatures (Chovancek et al. 2021), which can be well explained by the leaks of H^+^ through the thylakoid membrane (Bukhov et al. 1999; Havaux et al. 1996).

Although the proton leakage of the thylakoid is not a typical response associated with the salt stress, there are several papers indicating the leakage as a result of oxidative damage or stress-associated structural changes in the chloroplast (Richter et al. 2004; Sun et al. 2011). Moreover, the resistance to salt stress at the thylakoid level is associated with the stability of thylakoid membranes given by their lipid composition (Allakhverdiev et al. 1999; Sui et al. 2010). In our study, the values of MDA (Fig. 1) clearly indicated the oxidative damage in salt-sensitive genotypes, but not in salt tolerant.

In stress conditions, the over reduction of the PSI acceptor side is associated with excessive photoreduction of molecular oxygen, and ROS are produced (Asada 1999), causing oxidative damage with negative consequences on photosynthetic performance and photoprotection (Takagi et al. 2016). To avoid the over reduction of the electron transport chain, the proper regulation of linear electron transport is crucial, which requires the buildup of the transthylakoid proton gradient (Joliot and Johnson 2011). A low ECS_t_ in salt-stress exposed plants of susceptible genotypes in our experiment shows that the accumulation of H^+^ in thylakoid lumen was insufficient, which leads to a poor regulation of electron transport and over reduction of PSI acceptor side (Miyake et al. 2005; Chovancek et al. 2021). The related excessive production of reactive oxygen species may be responsible for the observed damage of photosynthetic components evident in chlorophyll fluorescence measurements as well as the decrease of chlorophyll content in leaves of susceptible wheat genotypes. In this respect, our results demonstrate that the noninvasive simultaneous measurements of chlorophyll fluorescence and electrochromic band shift using a low-cost MultispeQ sensor represents a reliable tool not only to identify the resistance or susceptibility of the genotypes to stress, but it can efficiently uncover the defects in the thylakoid membrane functions associated with the decrease of photosynthetic and photoprotective capacity of the leaves.

We also tested the use of the absorbance changes at 830 nm measured by the MultispeQ sensor in parallel with the chlorophyll fluorescence data. However, the P700 records in the actual setting were rather noisy, and the only useful information obtained was the maximum amplitude of the P700 signal, which can be applied as an indicator of the content of active PSI units (Schreiber et al. 1988; Brestic et al. 2015). Nevertheless, our results indicate (Fig. 5D) that the only information obtained is the decreased number of active PSI reaction centers in highly-stressed samples. As we know that there was a decrease of chlorophyll content in that samples, the decrease of PSI (as well as of the PSII found by fast chlorophyll fluorescence) is expected and, hence, the information has no particular value. Moreover, due to many records with excessive noise, the analysis of the values for individual genotypes could not be performed. Thus, despite the PhotosynQ platform analyzing the MultispeQ data provides the parameters related to the function and state of photosystem I, the quality of the measured signal was insufficient to provide useful data on the salt stress, and further improvements of the sensors or protocols are needed to obtain the valuable information on the P700 redox state using this sensor.

## Conclusion

The salt stress treatments led to an expected accumulation of sodium ion (Na^+^) and decreased potassium ion (K^+^) contents in leaves. The salinity led to an increase of soluble sugars in leaves, as well as increases of free proline content and MDA, with an apparent diversity of the responses between the genotypes. The evaluation of salt-treatment effects confirmed a higher tolerance to salt in MIR, GOB, and GYZ, whereas the FAT and ZIR were found as sensitive genotypes. The noninvasive measurements of photosynthesis-related parameters indicated genotype-dependent effects of salinity stress on the photosynthetic apparatus. The significant decrease of chlorophyll content (SPAD values) or adverse effects on photosynthetic functions at the PSII level (measured by the chlorophyll fluorescence parameters) was observed mostly in the two sensitive genotypes only. Although the information obtained by different fast noninvasive techniques was consistent, the correlation analyses identified the highest correlation of the noninvasive records with MDA, K^+^/Na^+^ ratio, and free proline content. The lower correlation levels were found for chlorophyll content (SPAD) and *F*_v_/*F*_m_ values derived from chlorophyll fluorescence. Performance index (PI_abs_) derived from fast fluorescence kinetics, and *F*_735_/*F*_685_ ratio correlated well with MDA and Na^+^ content. The most promising were the results of the linear electron transport (ETR_PSII_) measured by the MultispeQ sensor, in which we found a highly significant correlation with all parameters assessed.

Moreover, the noninvasive simultaneous chlorophyll fluorescence and electrochromic band shift measurements using this sensor can efficiently uncover the stress-induced alterations in the proton transport at the thylakoid membrane associated with a decrease of photosynthetic and photoprotective capacity of the leaves. Our results clearly demonstrate that the use of fast and low-cost portable sensors is not limited to identify the level of stress-induced damage in different plants, but the records can provide valuable mechanistic information on the specific stress effects and uncover the stress resistance mechanisms.
